# Anticipatory Saccades Towards the Future Consequences of One’s Actions – an Online Eye Tracking Study

**DOI:** 10.5334/joc.261

**Published:** 2023-02-10

**Authors:** Florian Gouret, Christina U. Pfeuffer

**Affiliations:** 1 Cognition, Action, and Sustainability Unit, Department of Psychology, Albert-Ludwigs-University of Freiburg, Freiburg, Germany; 2 Human-Technology Interaction, Department of Psychology, Catholic University of Eichstätt-Ingolstadt, Eichstätt, Germany

**Keywords:** Cognitive Control, Eye movements, Action and perception

## Abstract

When an action contingently yields a predictable effect, we form bi-directional action-effect associations that allow us to anticipate both the location and timing of our actions’ effects. This is evident in anticipatory eye movements towards the future effect’s location which are performed earlier when the effect’s delay is short rather than long. Such anticipatory eye movements reflect a proactive process of effect monitoring which prepares a comparison of expected and actual effects. Here, in two online eye tracking experiments, we manipulated effect locations (spatially compatible vs. incompatible in one half) and effect delays (short vs. long) to determine whether in-laboratory effects could be reliably replicated online using participants’ individual webcams. Extending prior research, we further compared irrelevant (Experiment 1) to relevant effects (response to effect feature; Experiment 2).

In contrast to prior in-laboratory studies, participants anticipatorily looked towards future effects above chance only when effects were relevant. Post-experiment questions suggested that online-participants intentionally ignore irrelevant information to optimize task performance. Nevertheless, replicating in-laboratory experiments, both for relevant and irrelevant effects, participants’ first saccade towards a future effect occurred earlier for the short rather than the long effect delay. Thus, we demonstrate that anticipatory eye movements reflecting a time-sensitive proactive effect monitoring process can reliably be assessed both in-laboratory as well as online. However, when investigating anticipatory saccade frequencies online, additional aspects like effect relevance have to be considered.

## Introduction

It is rather common that when we turn on our computer, we immediately look at the screen waiting for its response. As simple as this may appear, such eye movements towards locations at which we expect consequences of our actions inform us about how we control our actions. In the laboratory, using eye trackers with high temporal and spatial resolution, such anticipatory eye movements have been studied for several years (e.g., Gouret & Pfeuffer, 2021; Pfeuffer et al., 2016, 2022). Here, we assessed whether and under what conditions anticipatory eye movement measures relevant for the study of human action control could reliably be replicated online using participants’ own webcams. A reliable assessment of anticipatory eye movements even under suboptimal tracking conditions, for instance, online, is also a precondition for their future usage in the context of human-technology interaction settings.

Ideomotor theory (Elsner et al., 2002; Elsner & Hommel, 2001, 2004; Hommel, 2009; Hommel et al., 2001; James 1890/1981; Kunde, 2001; Sun et al., 2020; see e.g., Shin et al., 2010, for a review) postulates that when an action contingently yields the same effect, a bi-directional action-effect association is formed between action and effect (e.g., Dutzi & Hommel, 2009, Elsner & Hommel, 2001; Hommel et al., 2001; Kunde, 2001). Evidence for this notion comes, for instance, from the finding that participants respond faster when their key presses contingently produce spatially response-effect compatible visual effects (left key press –> visual effect on the left; e.g., 1^st^ half of the experiment) than when their actions produce spatially response-effect incompatible visual effects (left key press –> visual effect on the right; e.g., 2^nd^ half of the experiment). Such response-effect compatibility effects demonstrate that we anticipate our actions’ effects before executing the corresponding action (see, e.g., Hoffmann, 2003; Koch & Kunde, 2002; Pfister et al., 2010; 2014; Pfister, Janczyk, et al., 2014; Wirth et al., 2015). This effect anticipation allows us to retrieve the associated action and thus select the appropriate action to realize the intended effect (strong version of ideomotor theory; e.g., Elsner & Hommel, 2001; Hommel et al., 2001; Kunde, 2001).

Importantly, anticipating our actions’ effects does not only allow us to select appropriate actions that will produce these effects. It also starts a process of proactive effect monitoring in preparation for later comparing expected and actual effect as evident in anticipatory saccades (e.g., Gouret & Pfeuffer, 2021; Pfeuffer et al., 2016, 2022). When the location of visual effects is predictable due to prior learning experiences, in a blank screen interval between a manual response target offset and effect onset, participants look more often towards the future effect’s location than in the opposite direction (saccade-effect congruency, SEC, effect; e.g., Gouret & Pfeuffer, 2021; Pfeuffer et al., 2016; 2022). This was the case both when action-effect contingencies persisted for a large number of trials (1^st^ vs. 2^nd^ half of the experiment; e.g., Pfeuffer et al., 2016, 2022) and when action-effect contingencies only persisted for sequences of several trials before switching, that is, after few prior action-effect learning instances (Gouret & Pfeuffer, 2021).

Interestingly, participants do not only anticipate the effects’ future location but also the time at which their actions’ effects will appear. When effect delays were long rather than short, participants responded slower (Dignath et al., 2014, 2017). This has been taken as evidence that anticipating a further delayed effect takes longer, that is, the timing of effects is included in the action-effect association as well as the effect anticipation. Specifically, participants also anticipated when the consequences of their actions would occur regardless of what these consequences were (Dignath & Janczyk, 2017).

The timing of future effects is especially important when one tries to proactively monitor effects. Correspondingly, it has been shown that anticipatory saccades do not only reflect the location but also the timing of anticipated future effects (Gouret & Pfeuffer, under review/preprint; see also Kenward, 2010, for similar prior findings in infants). When left/right manual responses predictably caused visual effects after a short/long effect delay, participants’ first saccade towards the future effect’s location (effect-congruent) was performed earlier/later corresponding to the effect delay. This was the case even when action-effect delay mappings were only consistent within short trial sequences (Gouret & Pfeuffer, under review/preprint). That is, anticipatory saccade latencies (i.e., proactive effect monitoring) were adapted to the timing of future effects after few learning instances.

Importantly, processes of proactive effect monitoring and corresponding anticipatory saccades, so far, have only been studied with in-laboratory eye trackers with high temporo-spatial resolution. For an application to a larger range of studies and to confirm the reliability of anticipatory saccades as a measure of proactive effect monitoring under suboptimal eye tracking conditions, it is essential to assess to what degree anticipatory saccades can also be observed with eye trackers with low temporo-spatial resolution. Especially, demonstrating that anticipatory saccade frequency and latency effects can also reliably be measured with online eye trackers using participants’ own webcams is interesting for a future usage in applied contexts. For instance, anticipatory saccades occurring during the usage of various interfaces assessed via eye tracking based on the users own webcam could provide new insights into human-technology interaction or even be used as pieces of information guiding interface responses.

Regarding behavioural experiments, several comparisons of in-laboratory and online experiments have already been conducted (Del Popolo et al., 2022; Gosling et al., 2004; Innes et al., 2020; Reimers & Stewart, 2015; Semmelmann & Weigelt, 2016). There have also been first assessments of online eye tracking using participants own webcams as compared to in-laboratory experiments (e.g., Johansen et al., 2011; Semmelmann & Weigelt, 2018). Such prior studies demonstrated that online behavioural as well as online eye tracking experiments using participants’ own webcam provide rather reliable data. Here, we assessed whether anticipatory saccades occurring during goal-directed action control could also be reliably assessed via participants’ personal computers and webcams. Furthermore, we assessed the impact of effect relevance on anticipatory saccades to further understand the mechanisms of proactive effect monitoring in human action control.

To generally confirm the reliability of anticipatory saccade frequency and latency effects using webcam-based eye tracking of relatively low temporo-spatial resolution, we conducted two online eye tracking experiments (building on the paradigm of Pfeuffer and colleagues (Gouret & Pfeuffer, 2021; Pfeuffer et al., 2016, 2022). In both experiments, participants had to respond to a forced choice, response repeat/switch target with a left/right keypress and correct responses predictably led to a visual effect on the left/right side of the screen (i.e., at a spatially response-effect compatible/incompatible location; 1^st^ vs. 2^nd^ half of the experiment). Furthermore, one response caused an effect after a short delay, whereas the other response caused an effect after a long delay. We used participants’ webcams to assess their eye movements between target offset and effect onset. In Experiment 1, like in prior studies (e.g., Gouret & Pfeuffer, 2021; Pfeuffer et al., 2016, 2022), effects were irrelevant. In Experiment 2, extending prior in-laboratory studies, we rendered effects relevant to investigate the impact of effect relevance on anticipatory saccades in the online eye tracking setting.

We hypothesized that we would be able to replicate both the SEC effect in anticipatory saccade frequencies (e.g., Gouret & Pfeuffer, 2021; Pfeuffer et al., 2016, 2022) as well as the influence of effect delay on anticipatory saccade latencies (Gouret & Pfeuffer; under review/preprint) online using participants’ own webcams. Furthermore, based on the findings of Experiment 1 using irrelevant effects, we presumed that effect relevance would boost both frequency and latency effects in anticipatory saccades.

## Experiment 1

In Experiment 1, participants’ actions produced predictable but irrelevant visual effects.

### Method

#### Participants

The central effect we wished to replicate online in Experiment 1 was the influence of effect delay on saccade latencies. We simulated the sample size required to observe an influence of effect delay (200 ms vs. 800 ms) on saccade latencies based on a corresponding linear mixed model of the data of Gouret and Pfeuffer (under review/preprint) as suggested by Kumle et al. (2021). This sample size simulation suggested that ten participants would be required to observe an influence of effect delay on saccade latencies with at least 80% power at α = .05. To enable future comparisons with our prior experiments and to account for a potentially reduced effect in an online setting, we decided upon a planned sample size of roughly 24 participants. Note that the difference between the observed frequency of anticipatory saccades towards the future effect rather than in the opposite direction as compared to chance has consistently been observed to show large effects (e.g., Gouret & Pfeuffer, 2021; Pfeuffer et al., 2016, 2022) that a sample of 24 participants is sufficient to replicate as well.

Twenty-four participants completed the online eye tracking experiment (6 males, 18 females, mean age = 34.1 years, SD = 9.8, 3 left-handed, 1 Psychology student, 23 workers). Participants were recruited via Prolific and were native English speakers, had normal or corrected-to-normal vision, and a Prolific approval rate of at least 95%. Only participants who had a computer or laptop with a screen of at least 15” as well as a webcam could join the experiment. Furthermore, the data of participants who aborted the experiment, who switched tabs during the task, whose sampling rate was below 25 samples/second or who could not be tracked with at least 80% accuracy in an initial tracking check, or who performed at less than 70% accuracy in an initial practice (see Design and Procedure) was not considered. For these participants, the experiment was automatically aborted and they were replaced with new participants. Moreover, participants whose mean error rates exceeded the sample average by more than three standard deviations were also excluded and replaced. No participant was excluded due to this criterion. All participants were naïve to the purpose of the experiment, provided informed consent at the beginning of the online experiment, and received 7.50 GBP as compensation for their participation. Participants who could not complete the experiment received compensation in accordance with Prolific guidelines. Thirteen additional participants started the experiment, but failed to meet the technical requirements (e.g., low sampling rate or insufficient tracking accuracy in a first check). For these participants, the experiment was aborted with a corresponding notification and they were compensated for the time they spent. The study was conducted in agreement with the Declaration of Helsinki and the guidelines set by the local ethics committee.

#### Stimuli and Apparatus

Participants took part at home on their own computer or laptop. The experiment was programmed in JsPsych 6.3.1 and eye movements were tracked using the Webgazer library (Papoutsaki et al., 2017) and participants’ personal webcam. At the beginning of the experiment, participants used a bank card to resize a rectangle on the screen, so that stimulus size was the same for all participants. Moreover, an initial tracking check assessed the sampling rate and the accuracy with which participants’ eye movements could be tracked. Throughout the experiment, before each block, 9 points calibration and validation were performed and repeated up to 5 times in case tracking accuracy did not reach at least 80%. The index finger of participants’ left and right hand rested on the keys S and L. Throughout the experiment, the screen background was black.

#### Design and Procedure

Participants were first asked a set of demographic questions. Then, they conducted a practice of the task (20 trials without effects) in which participants needed to reach 70% accuracy (one retry possible). Subsequently, the main experiment consisting of 8 blocks of 50 trials ensued and was followed by post-experiment questions regarding encountered problems or disturbances as well as the presumed purpose of the study and observed regularities. Participants’ eye movements and performance measures were only recorded during the main experiment.

**Figure 1:**
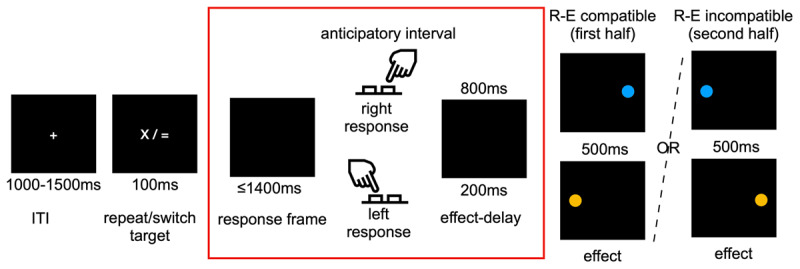
Trial structure: A repeat/switch target (100 ms) was followed by a blank screen response frame (1400 ms). Participants’ correct left/right responses contingently produced visual effects (orange vs. blue circle) on the spatially response-effect (R-E) compatible/incompatible left/right side of the screen (1^st^ vs. 2^nd^ half of the experiment) after a short versus long effect delay (200 vs. 800 ms). Each response was mapped to one effect colour and one effect delay (e.g., left response –> blue circle, 200 ms; right response –> orange circle, 800 ms). Trials were separated by a jittered intertrial interval (ITI). We assessed eye movements in the anticipatory interval between target offset and effect onset.

Each trial of the main experiment started with the presentation of a white fixation cross (0.6°) in the middle of the screen for a jittered inter-trial interval (ITI) ranging between 1000 ms and 1500 ms. The fixation cross was then replaced by a target (100 ms, 0.6°) indicating whether to press the left (S) or right (L) key. For every first trial of a block, the target was an arrow pointing either to the left or to the right. The direction indicated the first key to press. In the other trials of each block, the target was either an “=”, indicating to press the same key as in the previous trial (same response/response repeat), or a “x” indicating to press the opposite key as compared to the previous trial (opposite key/response switch). The reference was always the correct response on the previous trial. That is, after an error, participants had to select the same/opposite response according to the response they had not executed on the previous trial. These response repeat/switch forced choice targets were chosen to ensure that only action-effect associations and not target-effect associations could influence effect anticipation and corresponding anticipatory saccades (see Pfeuffer et al., 2022, for evidence that anticipatory saccade measures do not differ between left/right and response repeat/switch forced choice targets). The target was followed by a blank screen response frame of up to 1400 ms (i.e., response limit 1500 ms; see Figure 1 for the trial structure). Target onset was set as the zero mark for both manual reaction times and saccade latencies even though only saccades starting after target offset were analysed.

Correct responses were followed by a blank screen action-effect interval lasting for 200 ms or 800 ms (effect delay; e.g., left response – 800 ms, right response – 200 ms). After the action-effect interval, a visual effect (orange vs. blue circle, 500 ms, 1.5°) appeared 14° to the left/right of the screen centre. Per participant, one response was contingently associated with one effect colour and effect delay and the mappings were counterbalanced across participants. In one half of the experiment (e.g., blocks 1–4), effects appeared at locations spatially response-effect compatible with the manual response (e.g., left response –> effect on the left), in the other half of the experiment (e.g., blocks 5–8), effects appeared at locations spatially response-effect incompatible with the manual response (e.g., left response –> effect on the right). Response-effect compatibility order was counterbalanced across participants. After the effect was presented, the next trial began.

In case of an incorrect, premature, or omitted response, no effect was presented. Instead corresponding feedback was displayed in red in the centre of the screen (“too early!” for premature responses, “error!” for incorrect responses, and “too slow!” for response omissions; duration: 1000 ms) and the trial was aborted.

At the end of each block of the main experiment, participants received feedback about their performance (i.e., number of errors, omitted or premature responses). They were then reminded to respond as fast and accurately as possible.

Participants were not informed about contingencies between their responses and effect delays, effect positions, or effect colours. Moreover, no instruction or information regarding eye movements was given. Thus, any saccade performed during the assessed anticipatory interval between the target offset and effect onset can be considered as spontaneous and uninstructed.

### Result

All statistical analyses were performed using R version 4.1.2. Practice trials, the first trial per block as well as trials with premature (<0.1%) or omitted responses (<0.1%) were excluded from all analyses. Linear mixed models (LMMs) were used to assess saccade latency and manual reaction time and generalized linear mixed model (GLMMs) were used to assess saccade-effect congruency and errors. (G)LMMs were computed with the lme4 (Bates et al., 2015), lmerTest (Kuznetsova et al., 2017), pbkrtest (Halekoh & Højsgaard, 2014), afex (Singmann et al., 2015), and emmeans (Lenth et al., 2018) packages. To create figures, we used the ggplot2 package (Wickham, 2016). For LMMs, we used the maximum likelihood estimation and the Satterthwaite (Kuznetsova et al., 2017) method to assess p values for model selection and restricted maximum likelihood estimation and the Kenward-Roger (Kenward & Roger, 1997) approximation for denominator degrees of freedom to examine the final model. For GLMMs, we used maximum likelihood estimation and binomial link functions (bobyqa optimizer, 1,000,000 iterations) and p values were estimated via asymptotic Wald tests. For GLMMs, we report odds ratios (Szumilas, 2010). Conditional *R^2^* values for each mixed model were computed using the r.squaredGLMM function of the MuMIn package (Nakagawa & Schielzeth, 2013; see also Nakagawa et al., 2017). Both of our two-level predictors were contrast coded (–1/1) such that estimates in the linear mixed model table (β) indicate the difference between one of the conditions and the grand mean.

The (G)LMMs of all dependent variables included the fixed effects response-effect compatibility (response-effect compatible vs. response-effect incompatible) and effect delay (short vs. long) as well as their interaction. For all dependent variables, we started with a random effects structure that included participant intercepts and by-participant random slopes for response-effect compatibility and effect delay. In case the (G)LMM did not converge, the model’s complexity was decreased. We first removed correlations among random slopes, then the by-participant random slopes of effects response-effect compatibility, and finally the by-participant random slopes of effect delay until the model converged without a singular fit or a negative Hessian eigenvalue.

#### Manual responses

##### Manual Reaction Time

**Figure 2:**
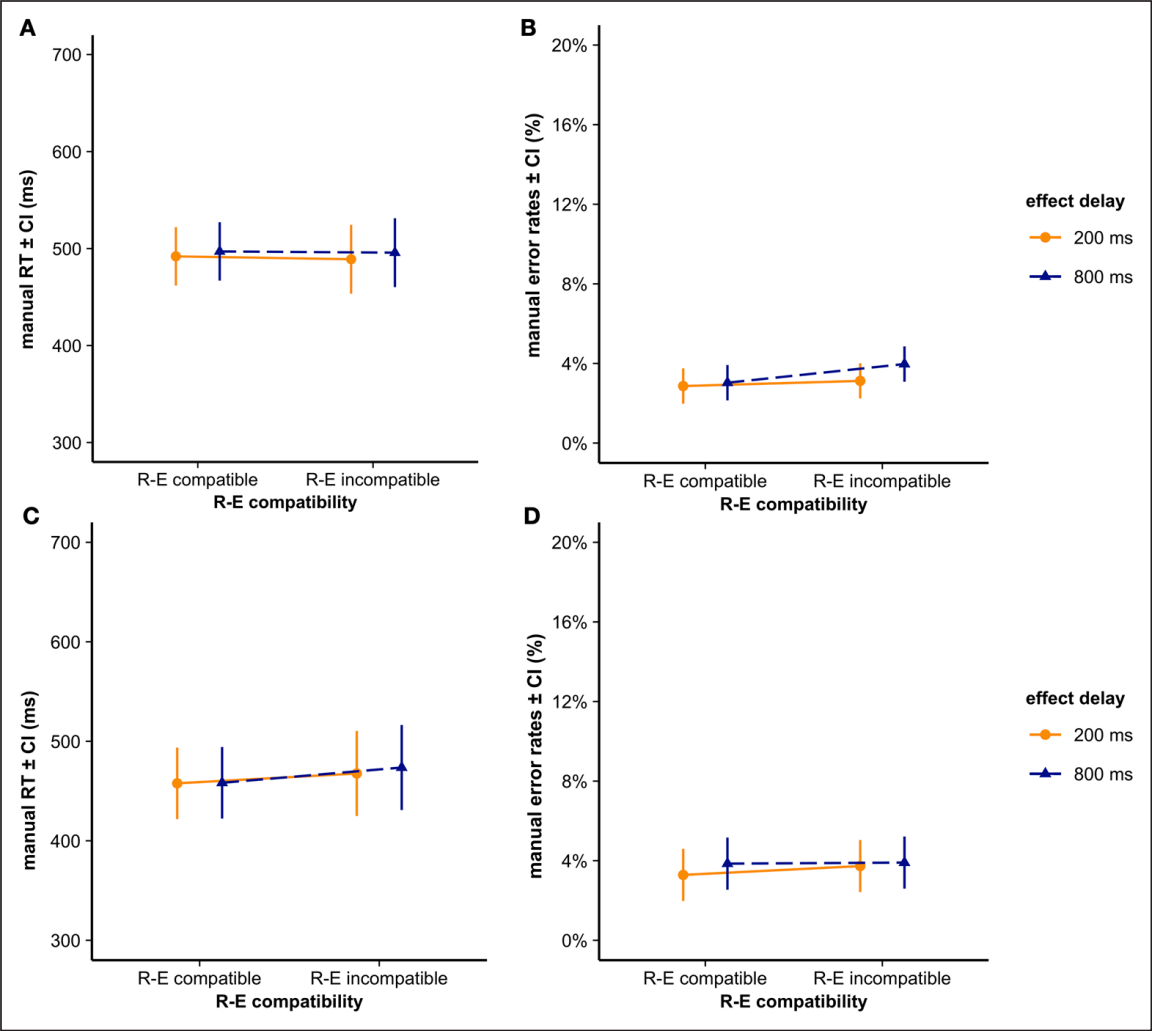
Manual reaction times (RTs) in **A)** Experiment 1 and C) Experiment 2 and error rates in **B)** Experiment 1 and D) Experiment 2 and displayed per response-effect (R-E) compatibility and effect delay condition. Error bars depict the 95% between-subject confidence interval of the mean.

For the analysis of manual reaction times (RTs), trials containing errors as well as trials with an RT deviating more than three SDs from their individual cell mean (i.e., outliers) were not considered. The mean RT was 494 ms (SD = 149 ms). The LMM fitting the manual RT included participant intercepts and by-participant random slopes of effect delay as random effects (see Figure 2B). No significant effect was observed, |*ts*| ≤ 1.40, *ps* ≥ .174 (see Table 1 for detailed results).

**Table 1:** Linear Mixed Model Results per Experiment: Manual Reaction Time.


	MANUAL REACTION TIME (RT)									

EXPERIMENTS	EXPERIMENT 1					EXPERIMENT 2				
	
*PREDICTORS*	*ESTIMATE*	*CI*	*SE*	*T*	*P*	*ESTIMATE*	*CI*	*SE*	*T*	*P*

Intercept	493.45	463.49–523.41	14.48	34.08	**<0.001**	464.34	428.40–500.29	17.33	26.79	**<0.001**

Response-effect compatibility	1.07	–1.68–3.81	1.40	0.76	0.446	–6.30	–9.24–3.37	1.50	–4.21	**<0.001**

effect delay	–2.95	–7.31–1.40	2.10	–1.40	0.174	–1.63	–6.69–3.43	2.44	–0.67	0.504

Response-effect compatibility × effect delay	0.43	–2.32–3.18	1.40	0.31	0.759	1.36	–1.58–4.30	1.50	0.91	0.364

**Model**										

N _Subject_	24					23				

Observations	8907					8445				

Marginal R^2^/Conditional R^2^	0.000/0.222					0.002/0.267				

Deviance	112412.343					107267.066				


*Note*: CI indicates the 95% confidence interval and SE refers to standard error. Converging model Experiment 1: RT ~ Response-effect compatibility * Effect delay + (Effect delay || Subject). Converging model Experiment 2: RT ~ Response-effect compatibility * Effect delay + (Effect delay || Subject).

##### Manual error rate

The mean error rate was 3.8% (SD = 2.1%). The GLMM fitting error (0 = correct, 1 = error) included participant intercepts and by-participant random slopes for response-effect compatibility and effect delay as random effect (see Figure 2A). No significant effect was observed, |*ts*| ≤ 1.23, *ps* ≥ .219 (see Table 2 for detailed results).

**Table 2:** Linear Mixed Model Results per Experiment: Manual Errors (0/1).


	ERROR									

EXPERIMENTS	EXPERIMENT 1					EXPERIMENT 2				
	
*PREDICTORS*	*OR*	*CI*	*SE*	*Z*	*P*	*OR*	*CI*	*SE*	*Z*	*P*

Intercept	0.03	0.03 – 0.04	0.00	–28.40	**<0.001**	0.04	0.03 – 0.05	0.01	–21.73	**<0.001**

Response-effect compatibility	0.91	0.79 – 1.06	0.07	–1.23	0.219	0.96	0.84 – 1.11	0.07	–0.50	0.616

effect delay	0.93	0.80 – 1.07	0.07	–1.01	0.312	0.95	0.83 – 1.09	0.07	–0.76	0.450

Response-effect compatibility × effect delay	1.05	0.94 – 1.17	0.06	0.86	0.388	0.97	0.88 – 1.07	0.05	–0.58	0.563

**Model**										

N _Subject_	24					23				

Observations	9365					8968				

Marginal R^2^/Conditional R^2^	0.005/0.047					0.001/0.056				

Deviance	2935.084					3199.983				


*Note*: CI indicates confidence interval and SE refers to standard error. Converging model Experiment 1: Error ~ Response-effect compatibility * Effect delay + (Response-effect compatibility | Subject) + (Effect delay|Subject). Converging model Experiment 2: Error ~ Response-effect compatibility * Effect delay + (Response-effect compatibility | Subject) + (Effect delay|Subject).

#### Anticipatory saccades

Only saccades occurring during the anticipatory interval between target offset and effect onset were assessed. In the present study, we determined saccade starting points and latencies by assessing between which consecutive samples the horizontal gaze position shifted by at least 2°. The data of the pre-movement sample was set as the saccade’s starting point and latency. Only the first saccade per trials was considered for the analysis of saccade-effect congruency and only the first effect-congruent saccade (i.e., directed towards the future effect) was considered for the analysis of saccade latencies. Note that this differs from prior in-laboratory studies on such anticipatory saccades in which all saccades per trial were considered for the analysis of saccade-effect congruency. The criteria for analysis were adapted to account for the temporally less precise saccade detection using participants’ individual webcams which sample at only around 30 Hz at maximum under ideal Browser conditions. In sum, 3,516 saccades were used for the saccade-effect congruency analysis and 2,791 saccades were considered for the saccade latency analysis. To infer tracking precision, we additionally assessed gaze sample distributions during target presentation. The mean dispersion of gaze samples in X direction during target presentation was 1.1° and the mean dispersion of gaze samples in Y direction was 1.6°. That is, gaze sample dispersion in a phase of the experiment in which participants should fixate one spot, the target location, was lower than the 2° saccade criterion we set to detect saccades.

##### Relative saccade frequency (saccade-effect congruency, SEC, scores)

The overall SEC score was computed dividing the number of first saccades per trial towards the future effect location (effect-congruent) by all saccades (including both effect-congruent and effect-incongruent saccades). A score higher than 50% (chance level) shows that participants anticipated their own actions’ consequences and moved their eyes towards them in anticipation, that is, proactively monitored their actions’ effects.

We thus, first conducted two one-sample t-tests to assess participants’ overall SEC scores. These one-sample t-tests showed that participants’ mean SEC was significantly greater than 50% in the response-effect compatible condition, *t*(23) = 3.12, *p* = .002, *d* = 0.64 (M = 55.8%, SD = 9.1%), but not in the response-effect incompatible condition, *t*(23) = 1.04, *p* = .155 (M = 52.4%, SD = 11.1%).

**Figure 3:**
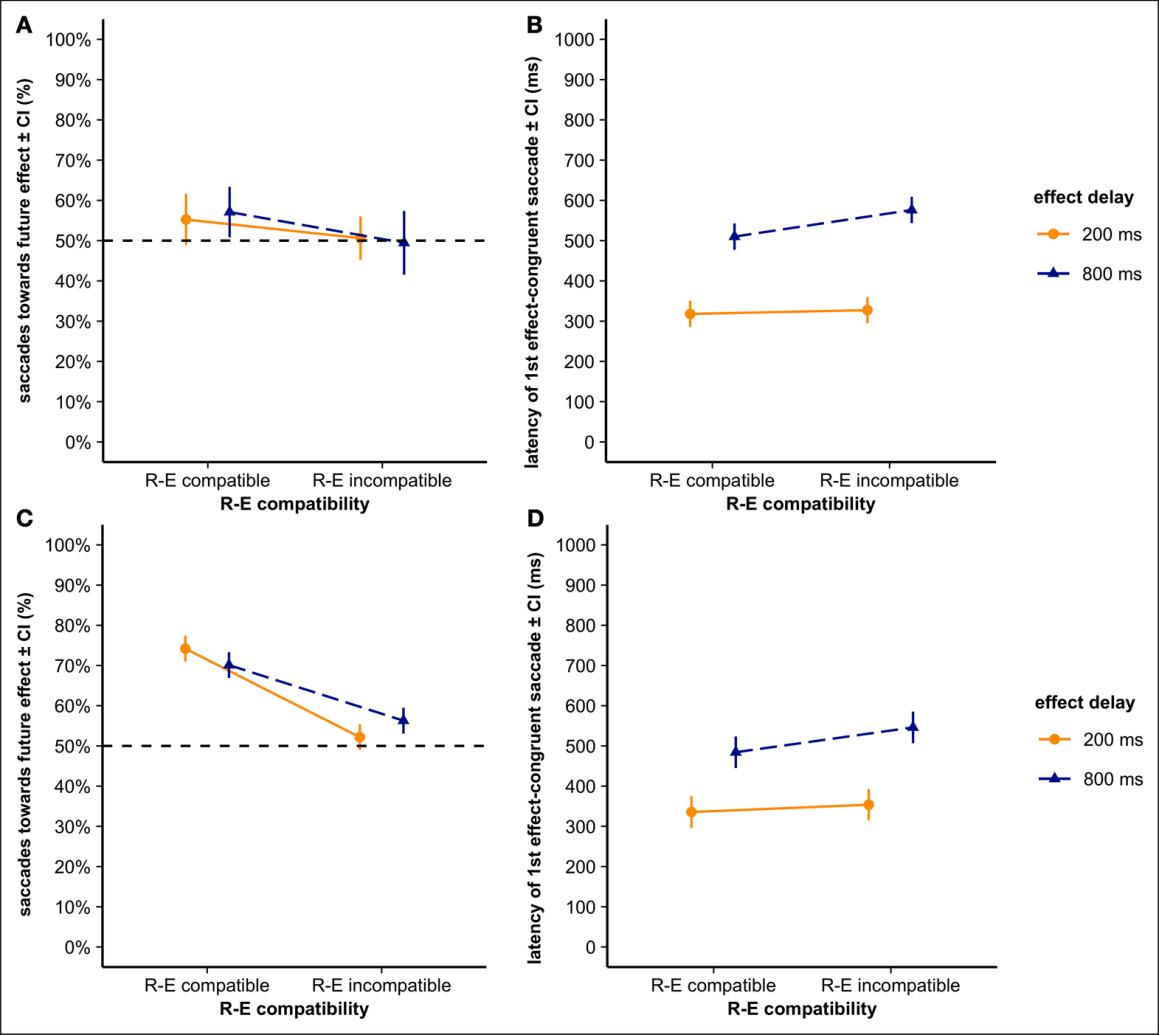
Saccade-effect congruency (SEC; saccades towards the effect/all saccades) score in **A)** Experiment 1 and **C)** Experiment 2 and latency of the first effect-congruent saccade in **B)** Experiment 1 and **D)** Experiment 2 displayed per response-effect (R-E) compatibility and effect delay condition. Error bars depict the 95% between-subject confidence interval of the mean.

The GLMM fitting saccade-effect congruency (0 = effect-incongruent, 1 = effect-congruent) included response-effect compatibility and effect delay slopes and participant intercepts as random effects (see Figure 3A). Response-effect compatibility reached significance, *z* = 3.51, *p* < .001, *OR* = 2.26. That is, participants performed more effect-congruent saccades when the future effect’s location was spatially response-effect compatible rather than response-effect incompatible to their response. Neither effect delay nor the interaction of response-effect compatibility and effect delay was significant, |*zs*| ≤ 0.88, *ps* ≥ .380 (see Table 3).

**Table 3:** Linear Mixed Model Results per Experiment: Saccade-Effect Congruency (SEC; 0/1).


	SACCADE EFFECT CONGRUENCY									

EXPERIMENTS	EXPERIMENT 1					EXPERIMENT 2				
	
*PREDICTORS*	*OR*	*CI*	*SE*	*Z*	*P*	*OR*	*CI*	*SE*	*Z*	*P*

Intercept	1.13	1.02 – 1.25	0.06	2.39	**0.017**	1.75	1.51 – 2.04	0.14	7.25	**<0.001**

Response-effect compatibility	1.13	1.06 – 1.21	0.04	3.51	**<0.001**	1.48	1.27 – 1.73	0.12	5.01	**<0.001**

effect delay	0.99	0.93 – 1.06	0.03	–0.21	0.837	1.01	0.91 – 1.13	0.06	0.17	0.864

Response-effect compatibility × effect delay	0.97	0.91 – 1.04	0.03	–0.88	0.380	1.1	1.02 – 1.17	0.04	2.66	**0.008**

**Model**										

N _Subject_	24					23				

Observations	3516					4260				

Marginal R^2^/Conditional R^2^	0.005/0.015					0.043/0.091				

Deviance	4827.508					5290.758				


*Note*: CI indicates confidence interval and SE refers to standard error. Converging model Experiment 1: SEC ~ Response-effect compatibility * Effect delay + (1 | Subject). Converging model Experiment 2: SEC ~ Response-effect compatibility * Effect delay + (Response-effect compatibility | Subject) + (Effect delay | Subject).

##### Saccade latency

For the saccade latency analysis, we only considered the first effect-congruent saccade per trial. The LMM on saccade latency included participant intercepts and by-participant random slopes for effect delay as random effects (see Figure 3B). The response-effect compatibility did not reach significance, *t* = –1.83, *p* = .080, |*2*β^1^| = 37.82 ms. Effect delay reached significance, *t* = –12.11, *p* < .001, |*2*β| = 220 ms. That is, participants’ first effect-congruent saccade was performed later for the long effect delay than for the short effect delay. Furthermore, effect delay and response-effect compatibility significantly interacted, *t* = 2.44, *p* = .015, |*2*β| = 28 ms (see Table 4). Participants showed larger latency differences between the short and the long effect delay in the response-effect incompatible, *t* = 11.05, *p* < .001, |*2*β| = 249 ms, as compared to the response-effect compatible condition, *t* = 9.30, *p* < .001, |*2*β| = 192 ms.

**Table 4:** Linear Mixed Model Results per Experiment: Saccade Latency.


	SACCADE LATENCY									

EXPERIMENTS	EXPERIMENT 1					EXPERIMENT 2				
	
*PREDICTORS*	*ESTIMATE*	*CI*	*SE*	*T*	*P*	*ESTIMATE*	*CI*	*SE*	*T*	*P*

Intercept	432.91	407.24 – 458.57	12.39	34.94	**<0.001**	428.89	397.26–460.52	16.13	26.59	**<0.001**

Response-effect compatibility	–18.91	–40.28 – 2.46	10.32	–1.83	0.080	–19.58	–41.21–2.05	11.03	–1.78	0.076

effect delay	–110.20	–129.06 – 91.33	9.10	–12.11	**<0.001**	–84.18	–106.52–61.83	11.40	–7.39	**<0.001**

Response-effect compatibility × effect delay	14.18	2.77 – 25.60	5.82	2.44	**0.015**	10.88	2.50–18.59	4.10	2.57	**0.010**

**Model**										

N _Subject_	24					23				

Observations	2791					3780				

Marginal R^2^/Conditional R^2^	0.126/0.130					0.111/0.171				

Deviance	39567.12					52181.490				


*Note*: CI indicates the 95% confidence interval and SE refers to standard error. Converging model Experiment 1: Saccade latency ~ Response-effect compatibility * Effect delay + (Response-effect compatibility || Subject) + (Effect delay || Subject). Converging model Experiment 2: Saccade latency ~ Response-effect compatibility * Effect delay + (Effect delay || Subject).

### Discussion

In Experiment 1, we aimed to replicate the SEC effect in anticipatory saccade frequencies (e.g., Gouret & Pfeuffer, 2021; Pfeuffer et al., 2016, 2022) as well as the influence of effect delay on anticipatory saccade latencies (Gouret & Pfeuffer; under review/preprint). Participants had to respond to a forced choice, response repeat/switch target with a left/right key press. Correct responses were predictably followed by an effect at a spatially response-effect compatible or response-effect incompatible location. One response caused a visual effect after a short effect delay of 200 ms, whereas the other response caused a visual effect after a long effect delay of 800 ms. Crucially, effects were irrelevant to participants’ task.

SEC scores were just above chance level in the response-effect compatible condition (i.e., participants looked towards future effects above chance), but did not differ from chance in the response-effect incompatible condition. This pattern is in stark contrast to the results of prior in-laboratory studies (e.g., Gouret & Pfeuffer, 2021; Pfeuffer et al., 2016, 2022). Although SEC effects were previously observed to be smaller in the response-effect incompatible than the response-effect compatible condition (e.g., Gouret & Pfeuffer, 2021; Pfeuffer et al., 2022), SEC effects in prior in-laboratory studies were, on average, substantially above chance (>70%). Moreover, it is unlikely that the lack of an SEC effect in the response-effect incompatible condition and the very subtle SEC effect in the response-effect compatible condition can be attributed to the less precise eye tracking methodology. The webcam-based eye tracking might have prevented us from registering saccades as precisely, but this should not have selectively favoured the detection of effect-incongruent saccades (i.e., saccades away from the future effect).

Thus, we concluded that aspects of the online setting itself might have had an effect on participants. A possible explanation for the difference in result patterns regarding the SEC effect online as compared to in-laboratory was found in participants’ answers to our general post-experiment questions. When asked about the usage of any strategy to complete the task, participants reported that they tried their best to focus their eyes on the middle of the screen where the target would appear and not look anywhere else (e.g., on the irrelevant effects). This aligns with the observed data patterns. Participants, overall, did not perform many eye movements away from the target area. As such, they might have suppressed anticipatory saccades towards the future effect. This strategy might have been the result of the instructions given at the beginning of the experiment. Both in-laboratory and online, participants were asked to always respond as fast and accurately as possible. However, although errors during the testing phase were not an issue either in-laboratory or online, committing too many errors during a preceding training phase was a reason for premature exclusion and, correspondingly, reduced compensation in the online experiment in contrast to the prior in-laboratory experiments to ensure compliance with instructions. This might have led to a strategy in online participants which optimized on-target attention at the cost of attending to the (irrelevant) effects.

Nevertheless, although we only found a subtle SEC effect in the response-effect compatible condition, strong saccade latency effects were replicated (Gouret & Pfeuffer, under review/preprint). That is, participants performed their first effect-congruent saccade substantially later when the effect delay was long rather than short. Interestingly, the magnitude of this effect was larger than the one reported in Gouret & Pfeuffer (under review/preprint). This first confirms that the influence of effect delay on saccade latency could be replicated online even when using participants’ individual webcams. That such a strong effect was observed even though few effect-congruent saccades were performed further attests to the reliability of the saccade latency effect itself which does not seem to be crucially dependent on the exact experimental setting or the number of saccades included in the analysis. Moreover, it indicates that even when participants try to prevent themselves from performing anticipatory saccades towards their actions’ future effects, saccade latency is a reliable indicator of effect anticipation and proactive effect monitoring – much more so than the frequency of effect-congruent saccades.

The aim of Experiment 1 was to replicate two in-laboratory findings online: The SEC effect in saccade frequencies and the influence of effect delay on saccade latency. Although substantial differences in saccade latencies were found according to effect delay, saccade frequencies were barely affected by the future effect position and only differed from chance in the response-effect compatible condition. Based on participants’ reports, we concluded that they perceived the effects as irrelevant distractions due to the online instructions. Thus, we conducted a second experiment in which we made effects relevant to assess whether stable SEC effects comparable to in-laboratory settings could be observed online under these conditions.

## Experiment 2

Experiment 2 was the same as Experiment 1 except that effects were rendered relevant to participants’ task. That is, participants now saw a number within the coloured effect circle and had to respond to it in case it was a 3.

### Method

#### Participants

Sample size considerations were the same as in Experiment 1. Thus, a sample of ten participants should have sufficed to replicate the influence of effect delay on saccade latencies. However, to reach a sample size that allows for comparisons with our prior studies as well as Experiment 1, we aimed for a sample size of roughly 24 participants.

Participant inclusion criteria were the same as in Experiment 1. Like in Experiment 1, 24 new participants with full datasets were recruited via Prolific, but one participant indicated that their data could not be used in a corresponding post-experiment question. Thus, 23 participants were included in the final sample (14 males, 9 females, mean age = 33.1, SD = 10.1, 4 left-handed, all of them working). Nineteen additional participants started the experiment but failed the technical requirements. They were compensated for the time they spent in the experiment. No participants who completed the experiment were excluded due to high error rates. All participants were naïve to the purpose of the experiment, provided informed consent at the beginning of the online experiment, and received 7.50 GBP as compensation for their participation. The study was conducted in agreement with the Declaration of Helsinki and the guidelines set by the local ethics committee.

#### Stimuli and Apparatus

Stimuli and apparatus were the same as in Experiment 1.

#### Design and Procedure

The design was the same as in Experiment 1 with one exception. Whenever participants produced an effect, a random number (1–9, 1°, allocated to trials equally frequently across the entire experiment) was presented in the centre of the effect, that is, a feature of the effect became response relevant. In case this number was a three, participants were to respond by pressing the space bar. Effects were still presented for 500ms, but participants could respond for 1500ms (i.e., 1000ms into the following ITI). Participants did not receive trial-wise feedback on their responses/non-responses to the effect. At the end of a block, however, they were informed about the percentage of trials with an effect they had correctly responded/not responded on.

### Result

Trial exclusions and analyses were the same as in Experiment 1 (premature responses: <0.1%, response omissions: <0.1%). Participants correctly responded to the effect number 95.0% of trials with a mean RT of 690 ms (SD = 166 ms). We followed the same analysis strategy as in Experiment 1 and reduced the random effects structure accordingly in case a model did not converge without a singular fit or negative Hessian eigenvalues.

#### Manual response

##### Manual Reaction Time

The mean RT was 464ms (SD = 160ms). The LMM fitting manual RTs included participant intercepts and by-participant random slopes for response-effect compatibility and effect delay as random effect (see Figure 2C). response-effect compatibility was significant, *t* (23) = 4.21, *p* < .001, |*2*β| = 12.60 ms. Participants responded faster in the response-effect compatible condition than in the response-effect incompatible condition. Effect delay and the interaction of response-effect compatibility and effect delay did not reach significance, |*ts*| ≤ 0.91, *ps* ≥ .364 (see Table 1).

##### Manual Error rate

The mean error rate was 4.6% (SD = 3.0%). The GLMM fitting errors included participant intercepts and by-participant random slopes for response-effect compatibility and effect delay as random effect (see Figure 2D). As in Experiment 1, no significant effects were observed, |*zs*| ≤ 0.76, *ps* ≥ .450 (see Table 2).

#### Anticipatory saccades

Saccade selection criterion were the same as in Experiment 1. Only saccades occurring within the anticipatory interval (target offset to effect onset) fulfilling the inclusion criteria were considered. A total of 4,260 saccades were used for the saccade-effect congruency analysis and 3,780 saccades were considered for the saccade latency analysis. Again, to infer tracking precision, we additionally assessed gaze sample distributions during target presentation. The mean dispersion of gaze samples in X direction during target presentation was 0.7° and the mean dispersion of gaze samples in Y direction was 1.5°. That is, gaze sample dispersion in a phase of the experiment in which participants should fixate one spot, the target location, was lower than the 2° saccade criterion we set to detect saccades.

##### Relative saccade frequency (SEC effect)

We first conducted two one-sample t-tests to assess participants’ overall SEC scores. These one-sample t-tests showed that participants mean SEC were significantly greater than 50% in the response-effect compatible condition, *t*(22) = 11.4, *p* < .001, *d* = 2.38 (M = 74.8%, SD = 10.4%), as well as in the response-effect incompatible condition, *t*(22) = 9.01, *p* < .001, *d* = 1.89 (M = 66.9%, SD = 8.9%).

The GLMM fitting saccade-effect congruency (0 = effect-incongruent, 1 = effect-congruent) included participant intercepts and by-participant random slopes for response-effect compatibility and effect delay (see Figure 3C). Response-effect compatibility reached significance, *z* = 7.25, *p* < .001, OR = 2.96. Participants performed more effect-congruent saccades when the future effect’s location was spatially response-effect compatible rather than incompatible with their response. Moreover, the interaction of response-effect compatibility and effect delay was significant, *z* = 2.66, *p* = .008, OR = 2.20 (see Table 3 for detailed results). Participants did not show larger SEC differences between the short and long effect delay in the response-effect compatible, *z* = 1.52, *p* = .128, OR = 0.20, or in the response-effect incompatible condition, *z* = 1.29, *p* = .198, OR = 0.17. The interaction emerged due to opposing patterns. Effect delay did not yield a significant influence, *z* = 0.17, *p* = .864, OR = 2.02.

##### Saccade latency

Again, only the first effect-congruent saccade performed per trial was considered. The LMM fitting participants’ first effect-congruent saccades’ latency included participant intercepts and by-participant random slopes for response-effect compatibility and effect delay as random effect (see Figure 3D). The response-effect compatibility effect was not significant, *t* = 1.78, *p* = .076, |*2*β| = 39 ms. Effect delay reached significance, *t* = 7.39, *p* < .001, |*2*β| = 168 ms. That is, participants’ first effect-congruent saccade was performed later for the long as compared to the short effect delay. Effect-delay and response-effect compatibility significantly interacted, *t* = 2.57, *p* = .010, |*2*β| = 22 ms (see Table 4). Participants showed larger latency differences between the short and long effect delay in the response-effect incompatible, *t* = 7.78, *p* < .001, |*2*β| = 192 ms, than in the response-effect compatible condition, *t* = 6.19, *p* < .001, |*2*β| = 149 ms.

### Discussion

In Experiment 2, we modified the design of Experiment 1 by changing the relevance of the visual effect following participants’ responses. In Experiment 2, effects were relevant as compared to Experiment 1 in which they were irrelevant to participants’ task. That is, in Experiment 2, participants’ actions produced a coloured circle with a number at its centre and participants had to pay attention to the number and respond to it if it was a 3.

In contrast to Experiment 1, participants looked more often towards the future effect than expected by chance in both the response-effect compatible and response-effect incompatible condition, replicating both the result patterns and magnitudes (at least on response-effect compatible trials) found in prior in-laboratory studies (e.g., Gouret & Pfeuffer, 2021; Pfeuffer et al., 2016, 2022). This result corroborates that effect relevance supports higher anticipatory saccade frequencies and allows for observing stable SEC effects online.

In line with Experiment 1 and a prior in-laboratory study (Gouret & Pfeuffer, under review/preprint), a saccade latency effect was again observed in Experiment 2. Participants performed their first effect-congruent saccade later when the effect delay was long rather than short. In size, this saccade latency effect appeared to be comparable to Experiment 1. This further emphasizes the reliability of anticipatory saccade latencies as an excellent indicator of effect anticipation and proactive effect monitoring under various experimental conditions.

## General discussion

Across two online eye tracking experiments, we addressed the question whether anticipatory saccades – specifically frequency and latency effects observed in prior in-laboratory studies (Gouret & Pfeuffer, 2021, under review/preprint; Pfeuffer et al., 2016, 2022) – could be replicated online using participants’ individual webcams. In both experiments, participants’ actions, after a response-specific short/long effect-delay, led to the appearance of a visual effect predictably located at a position spatially compatible/incompatible to their response. Effects were irrelevant to participants’ task in Experiment 1, whereas they were relevant in Experiment 2.

Anticipatory saccade frequency effects were replicated in Experiment 2, but only very subtly present and only in the response-effect compatible condition in Experiment 1. In in-laboratory studies, the SEC effect, was consistently found to be strong (Pfeuffer et al., 2016, 2022; Gouret & Pfeuffer, 2021; under review/preprint; comparable to its size in Experiment 2). In line with our reasoning for manipulating effect relevance, this suggests that, depending on task demands, participants can suppress anticipatory saccades (e.g., when target attention is deemed as more essential). From a theoretical perspective, this is a very interesting finding as it questions to what degree proactive effect monitoring can be considered an automatic process (see e.g. Moors & De Houwer, 2006, for a discussion of different theoretical positions on automaticity). Comparing the findings of Experiment 1 to prior in-laboratory studies, it appears that participants only perform a substantial number of anticipatory saccades when attentional task demands are relaxed. This also aligns with the observation that anticipatory saccade frequencies substantially differ between individuals. Furthermore, this means that proactive effect monitoring (or at least its expression in anticipatory saccades) might rely on a cognitive resource that is also required for task-related processing and/or attention.

Importantly, effect relevance appeared to overrule participants’ strategy to suppress anticipatory saccades. When effects were relevant, participants again performed anticipatory saccades towards the location of their actions’ future effects well above chance level. Furthermore, our findings suggest that effect relevance mainly modulates the occurrence/frequency but not latency of anticipatory saccades, as effect delay comparably affected saccade latencies in both experiments. This extends prior in-laboratory studies (Pfeuffer et al., 2016, 2022; Gouret & Pfeuffer, 2021; under review/preprint) and also allows for the methodological recommendation to use relevant effects (or effect features) when it is essential to generate a large number of anticipatory saccades and/or when participants might try to suppress anticipatory saccades, as it might, for instance, be the case in online settings.

Moreover, even when anticipatory saccades were infrequent and SEC effects were not observable in saccade frequencies, saccade latencies nonetheless reliably reflected that participants anticipatorily monitored the timing of their actions’ future effects. That is, we observed longer saccade latencies for long than for short effect delays irrespective of SEC effects (or effect relevance). Methodologically, this highlights anticipatory saccade latencies as the more sensitive and reliable measure of proactive effect monitoring that can be observed even under suboptimal eye tracking conditions (e.g., online participants’ using individual webcams).

From a theoretical perspective, these observations clearly differentiate between saccade frequency and saccade latency effects. Saccade latency effects can be found irrespective of SEC effects and, in contrast to SEC effects, they did not seem to be affected by effect relevance. This suggests that these effects could emerge due to different subprocesses of proactive effect monitoring. This finding is also in line with prior studies on effect-generating actions which suggest that spatial and temporal features of an action’s future effect might independently be associated with the action and/or be anticipated independently (e.g., Dignath & Janczyk, 2017; Pfister et al., 2017). Building on the present findings and assessing whether there are multiple subprocesses of proactive effect monitoring will further increase our understanding of proactive effect monitoring and goal-directed action control.

Finally, our findings demonstrate that anticipatory saccades are a reliable measure of proactive effect monitoring even under suboptimal eye tracking conditions, for instance, during webcam-based online eye tracking. Our findings illustrated that an average webcam and computer, resulting in sampling rates below 30 Hz, are sufficient to assess anticipatory saccades. First, this replication under adversarial conditions speaks to the reliability of anticipatory saccade latency effects and (for relevant effects) anticipatory saccade frequency effects. Second, it broadens the range of settings in which anticipatory saccades can be assessed. On the one hand, they can be used in basic research on human action control even when one does not have an eye tracker with high temporo-spatial resolution at hand. On the other hand, that anticipatory saccades (i.e., proactive effect monitoring) can also be assessed online with participants’ webcams at below 30 Hz, opens up the chance of using anticipatory saccades as a measure in applied context, especially human-technology interaction settings, in the future.

## Data accessibility statements

The data as well as experiment files and syntaxes are publicly available on OSF: https://doi.org/10.17605/OSF.IO/AVFBX.

## Footnotes

Note that, given our contrast coding, β represents the difference between one condition and the grand mean. When comparing two conditions as in the reported analyses, 2β thus describes the size of the difference between conditions, that is, the effect size.
